# FAIR-SMART expands access to supplementary materials for research transparency

**DOI:** 10.1371/journal.pbio.3003428

**Published:** 2025-10-09

**Authors:** Chih-Hsuan Wei, Robert Leaman, Po-Ting Lai, Don Comeau, Shubo Tian, Zhiyong Lu

**Affiliations:** Division of Intramural Research, National Library of Medicine (NLM), National Institutes of Health (NIH), Bethesda, Maryland, United States of America; University of Bath, UNITED KINGDOM OF GREAT BRITAIN AND NORTHERN IRELAND

## Abstract

Supplementary materials accompanying scientific articles are critical components of biomedical research, offering detailed datasets, experimental protocols, and extended analyses that complement the main text. These materials play an important role in enhancing transparency, reproducibility, and scientific impact by providing in depth analyses and the details necessary for reproducing experiments. However, the lack of consistent and standard formats has limited the access to supplementary materials in scientific investigations. In response, we propose a novel system aimed to enhance FAIR access to Supplementary MAterials for Research Transparency (FAIR-SMART). Specifically, we first aggregate supplementary files in a single location, standardize them into structured and machine-readable format, and make them accessible via web APIs. Next, we employ advanced large language models to automatically categorize the tabular data, which represents over 90% of the textual content in supplementary materials, enabling precise and efficient data retrieval. By bridging the gap between diverse file types and automated workflows, this work not only advances biomedical research but also highlights the transformative potential of accessible supplementary materials in shaping the behaviors and decision-making processes of the scientific community. FAIR-SMART is freely available for supplementary materials data retrieval via its APIs: https://www.ncbi.nlm.nih.gov/research/bionlp/APIs/FAIR-SMART/.

## Abstract 2

Supplementary materials that accompany scientific articles enhance transparency, reproducibility and scientific impact. This study presents FAIR-SMART, a tool that enables computational access to these materials, improving findability, interoperability, and reuse, thereby improving FAIR access to supplementary data at scale.

## Introduction

**Fig 1:**
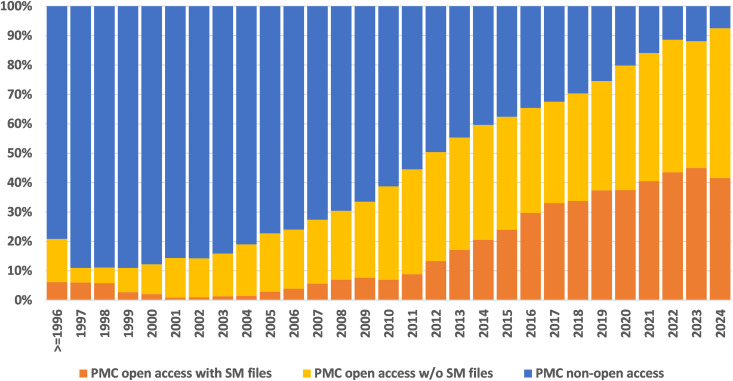
Proportion of PMC articles with supplementary materials available. The numerical data for this figure can be found in S1 Data.

Biomedical research produces a vast amount of textual data, including journal articles, clinical trial reports, patents, and supplementary materials. Supplementary materials (SM), often consisting of multiple files per article, play an important role in supporting the main findings. As shown in Fig 1, 27% of full-length articles in PubMed Central (PMC) include at least one SM file. In recent years, the percentage of articles containing SM files has increased significantly, with 40% of PMC articles published in 2023 containing at least one SM file. As such, this work specifically targets the subset of PMC open access articles that contain SM files. We also note from related research [1] a modest improvement in reusability, reflecting gradual progress in data sharing practices over time.

SM files help clarify details, add depth, and improve the transparency and reproducibility of research [2,3]. Top-tier journals often rely on SM data to provide essential details that cannot be fully covered in the main article, further prioritizing the reader’s time without sacrificing the integrity of the research [4]. This practice helps maintain the readability of articles while enabling researchers to access detailed datasets, experimental protocols, and additional analyses. For example, gene expression datasets, pathway analyses, and experimental protocols are frequently included in SM, making them critical resources for researchers who require detailed information beyond what is provided in the main text.

The widely-discussed reproducibility crisis in biomedical research [5] has highlighted the importance of providing detailed experimental methods and raw data to enable the replication of findings. SM often include raw datasets, detailed protocols, or comprehensive experimental methods that are essential for other researchers to reproduce biomedical studies and validate their results. A previous study [6] has shown that the inclusion of extensive SM is positively correlated with higher reproducibility, particularly when these materials contain well-documented data and clear methodological details. However, the inconsistent standardization of SM across journals poses challenges. For example, list-format data are stored in various file types (e.g., Word, Excel, PowerPoint, and tab-delimited text formats), which can complicate access and reduce the transparency that SM is intended to provide.

While SM hold a wealth of data, their analysis and integration into automated workflows and scientific investigations face several barriers. The lack of standardization in how SM are formatted and presented makes it difficult to automatically extract meaningful information. Specifically, there are three major challenges for the access of SM in scientific workflows:

1. **Diverse and unstructured file formats**: SM files are often presented in heterogeneous formats, including PDFs, Excel sheets, Word documents, and images. These formats lack uniformity and structure, requiring substantial effort for automated data extraction and integration.
2. **Limited searchability**: Existing literature search engines (e.g., PubMed or PMC) do not index the content of SM files, forcing researchers to manually inspect individual articles and their associated materials. This significantly increases the time and effort required to locate relevant data.
3. **Difficulty of data re-use:** The lack of a machine-readable format prevents the integration of SM data into automated workflows for machine learning, text and data mining. This limitation makes creating large datasets from SM across multiple articles infeasible, as researchers are unable to expand their efforts to large-scale analyses and meta-studies. As a result, the significant potential of these data-rich files remains largely untapped.

To address these challenges, we aggregate SM files in a single location, standardize their format using BioC XML and JSON [7], and make them accessible via web-based APIs. BioC is a structured and community-based framework for representing textual information and related annotations, and has been widely applied to the main text of articles to enable interoperability between different text mining systems [8] with great success. By converting these underutilized SM files into structured, machine-readable formats, we unlock new opportunities for text and data mining, enabling researchers to access the information in SM that was previously effectively hidden.

The proposed system successfully converted 99.46% of over 5 million SM textual files, overcoming challenges posed by diverse file types, including PDFs and spreadsheets. By transforming SM into machine-readable formats, the pipeline enables enhanced findability through semantic clustering and rich metadata, improved accessibility via programmatic web API access, greater interoperability with diverse biomedical tools, and increased reusability for large-scale analyses and reproducibility studies. As such, the pipeline enhances FAIR access to Supplementary Materials for Research Transparency (FAIR-SMART), unlocking the potential for utilizing SM files in large-scale data retrieval, annotation, and analysis. Our evaluations demonstrate that FAIR-SMART consistently outperforms PubMed, PMC full-text search, and the NLM Dataset Catalog (https://www.datasetcatalog.nlm.nih.gov/) in retrieving relevant datasets for representative biomedical queries. By standardizing SM in BioC-compliant formats and preserving structured tabular data, the pipeline ensures seamless integration into diverse workflows, supporting applications in genomics, proteomics, and other data-intensive fields. These efforts directly address the FAIR principles [9], significantly enhancing transparency, accessibility, and data-driven discovery in biomedical research. In this study, our focus is supplementary materials (SM) stored on the PMC website and does not include datasets hosted on external repositories.

## Results

This section highlights findings related to the distribution, structure, and utility of SM files, along with the benefits of integrating them into structured and automated workflows to improve data accessibility, transparency, and reproducibility. Our characterization of range of data types in FAIR-SMART notes their great depth and diversity.

### Distribution of the SM file formats

**Fig 2:**
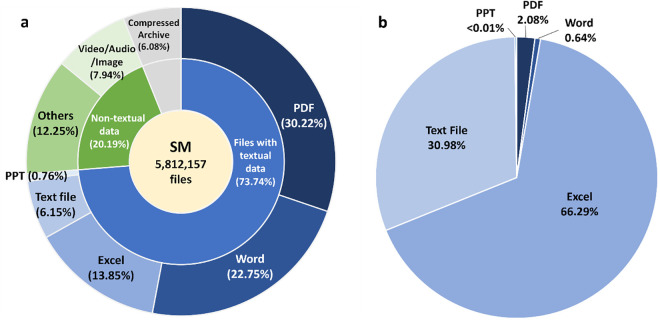
Across PMC Open Access articles, supplementary materials are heterogeneous in format, and structured tabular data are often supplied as textual files. **(a)** Distribution of SM file formats in the PMC Open Access subset. **(b)** Proportional of SM file to textual data sizes for each file format. The size distribution highlights the dominance of Excel and Text files in tabular data representation, despite their smaller contribution to total file counts. The textual data percentage for PowerPoint is less than 0.01%. The numerical data for this Figure can be found in S1 Data.

We collected the SM files from all articles in the PMC Open Access dataset and analyzed the distribution of their data formats. As illustrated in Fig 2a, the file formats with textual data —comprising PDF, Word, Excel, PowerPoint, and plain text files—make up 73.49% of the total SM files. Among these, PDF is the most frequent format, accounting for 30.22% of the total. Other textual formats include Word documents (22.75%), Excel files (13.85%), PowerPoint presentations (0.76%), and plain text files (6.15%). We further categorize the remainder of the SM files (20.19%) as non-textual data. The video/audio/image file (7.94%) is the major type. Other various types (e.g., *.sav as the binary IBM SPSS statistics data) occupied 12.25%. The reminded 6.08% are compressed files. The distribution of file types aligns with findings from previous studies [10–13].

A detailed breakdown of these file types is provided in S1 Table.

We further calculated the distribution of the textual data formats (i.e., PDF, Word, Excel, PowerPoint (PPT), and text files) based on their size (total file bytes). To visualize the proportion of the data size across different textual formats, we examined the proportional contributions of each format to the total textual data in Fig 2b. However, their date size is comparatively small due to their role in presenting summaries and reports, which are typically concise. Conversely, Excel and text files, while less common (13.85% and 6.15%, respectively), account for significantly larger proportions of the total textual data size shown in Fig 2b, at 66.29% and 30.98%. This discrepancy reflects their purpose as containers for raw and detailed datasets, such as extensive tables or computational results. PPT files are rare, making up less than 0.01%.

However, files with textual formats contain both free-text and tabular data. Inherently, Excel files are designed for managing structured information, and are fully convertible into tabular format, making them ideal for numerical and tabular data representation. Similarly, text files often stored in tab-delimited formats such as *.csv or *.tsv, are highly structured, with 87.25% (27.03% out of 30.98%) of their content easily transformable into tables, making them particularly suited for data sharing and analysis workflows. In contrast, PowerPoint slides are primarily created for presenting visual elements like bulleted lists and figures, have only 25% of their content suitable for tabular conversion. Analyzing tabular data within PDF files poses a significant challenge due to their focus on formatting and presentation. However, Word files, while frequently used for presenting mixed content such as text, figures, and tables, exhibit a relatively lower proportion of tabular data compared to Excel and text files, reflecting their flexible role in document presentation rather than structured data storage.

### Tabular data distribution and the content analysis

Based on our observations, 93.6% of textual files contain tabular data. To further explore the role and impact of SM tabular data in biomedical research, we conducted a detailed analysis of table content from a randomly sampled set of 10,000 PMC Open Access full-text articles, as summarized in Table 1. Among these articles, 3,688 contained at least one SM file, the total number of SM files in this set was 9,010. Although the number of files in the main text and SM are similar, the total size of the SM files (4,542,885,249 bytes) is more than 10 times greater than that of the main text (419,505,019 bytes). When focusing on tabular content, the number of tables in the main text is double that of the SM files (25,103 versus 12,268). However, the size of tabular content in the SM files is dramatically larger—142 times greater than that of the main text.

**Table 1:** Comparison of main-texts and SM files in sampled PMC articles.

Sampled data	Main-text	SM file
Total articles	10,000	
Total articles with SM files	3,688	
Total SM files	9,010	
Total # of tables	25,103	12,268
Total size of tables (# of bytes)	24,440,407	3,480,799,709

To further analyze the potential of tabular data in SM files for biomedical research, we used the ChatGPT API via the Azure platform to categorize the tables in our sample of 10,000 articles (the prompt is attached in Supplementary material). The total number of categories generated by ChatGPT for the 37,371 tables in both main-texts and SM files is over 5,000. We narrowed down over 5,000 categories to 40 by grouping them based on semantic similarity, determined through embeddings generated by a state-of-the-art language model and clustered using K-means. More details are provided in the “Method” section.

**Fig 3:**
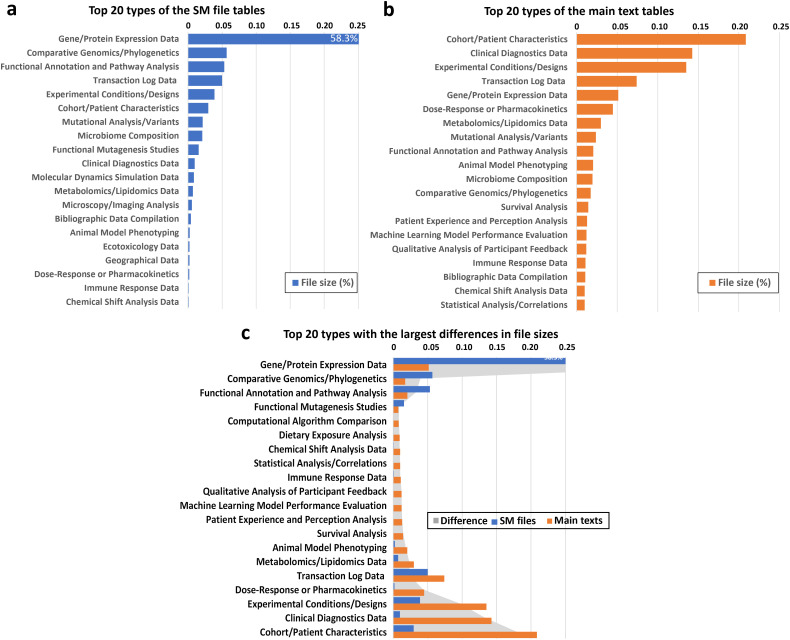
Distribution of table size across categories of SM files (a) and main texts (b). Panel **(c)** shows the top 20 table types with the largest size differences between SM files and main texts, highlighting their complementary roles in presenting detailed vs. summarized information. The numerical data for this Figure can be found in S1 Data.

We compared the counts of the 40 category clusters between the SM files and main texts. Of these, 15 categories out of the top 20 are shared between SM and main texts; however, their size distributions differ significantly, as illustrated in Fig 3. In SM files (Fig 3a), “Gene/Protein Expression Data” overwhelmingly dominate, contributing nearly 60% of the total file size, far surpassing other categories such as “Comparative Genomics/Phylogenetics,” “Functional Annotation and Pathway Analysis” and “Experimental Conditions/Designs.” In contrast, the distributions of the top files in article text (in Fig 3b) are similar, indicating that the file size of the tables in the main texts are much more consistent. Moreover, the leading category, “Cohort/Patient Characteristics,” accounts for nearly 20% of file size, followed by “Clinical Diagnostics Data,” “Experimental Conditions/Designs,” and “Transaction Log Data”. SM data tend to be more specialized and unevenly distributed. In contrast, the distributions in main texts are more balanced, with no single category dominating strongly. Moreover, the differing distributions underscore the distinct roles of SM (data-centric) and main texts (analysis-centric) in biomedical research.

To better understand the differences in table distributions, we compared the categories by total file sizes. The categories were sorted based on the disparities (Fig 3c) in SM files and main texts, showcasing the 20 categories with the greatest differences. Categories at the top indicate that the proportions of tables in SM files are significantly higher than in the main text, while categories at the bottom exhibit the opposite trend. This grey area illustrates the difference of the distributions between SM files and main texts. This analysis further highlights the diversity in table distributions between SM files and main texts.

### Bio-entities are enriched in SM data

We further analyzed the distribution of biomedical entities in the main texts and SM files of full-text articles. From the random sample of 10,000 full-text articles (in Table 1) in the PMC Open Access subset, 3,688 articles included supplementary textual files. We applied AIONER [14] to recognize entities within the SM files and extracted PubTator [15] annotations for the main texts using the PubTator3 API, as its back-end entity recognition tool is also AIONER.

Our analysis focused on six entity types registered in PubTator, as shown in S2 Table. The results revealed that SM data contained significantly more bio-entities per article compared to the main text. Notably, genes, cell lines, and variants appeared many times more frequently in SM files than in main texts. This observation aligns with the category distribution of SM tables illustrated in Fig 3a, where “Gene/Protein Expression Data” and “Comparative Genomics/Phylogenetics” emerged as the most popular categories, likely contributing to the abundance of genes and variants in the SM files. Additionally, these findings are consistent with prior studies [16–18] highlighting the genetic variant distribution in SM, further underscoring the critical role in capturing detailed biomedical information.

### Journal/scientific field/time analysis: Variations across disciplines and time

**Fig 4:**
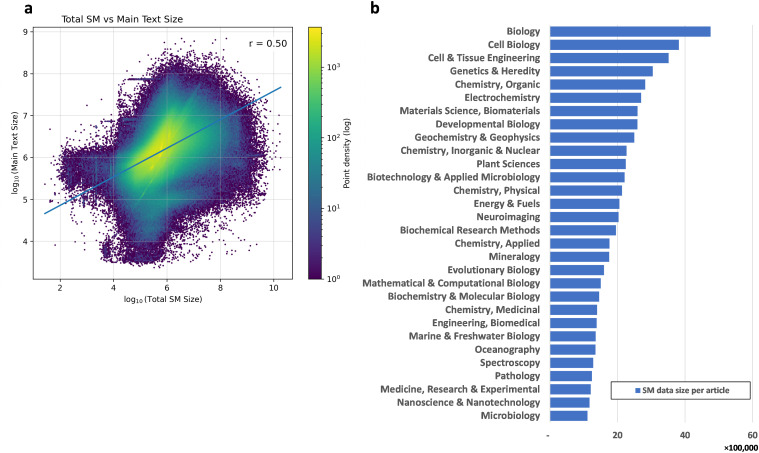
Supplementary material (SM) volume scales with main-text length and varies by discipline. **(a)** Correlation between the sizes of supplementary materials (SM) and main texts. **(b)** Distribution of SM data size per article across different journal fields, highlighting the variation in SM data usage among research areas. The numerical data for this Figure can be found in S1 Data.

To better understand how supplementary material (SM) data relate to primary research outputs, we examined the relationship between SM file size and the main text content. As shown in Fig 4a, there is a positive correlation (*r* = 0.50) between total SM size and main-text size across articles, suggesting that articles with more extensive main texts also tend to include larger volumes of SM data. This trend highlights the complementary nature of SM in providing detailed datasets, protocols, and extended results that support the core findings presented in the main text. Rather than serving as a substitute for main-text content, SM appears to expand upon it—offering additional depth and context that enhance transparency and facilitate reproducibility. This observation underscores the importance of ensuring that both main text and SM are accessible and well-structured for reuse in modern biomedical research workflows.

Beyond the year-over-year analysis, we also explored the relationship between SM usage and disciplinary focus. From the 254 categories registered in the Journal Citation Reports (JCR, https://jcr.clarivate.com), we identified 115 categories related to biological and biomedical fields, along with their corresponding journals. Fig 4b lists the top 30 categories with the highest number of SM files. The numbers of the SM data size per article of all other categories are listed in the S3 Table.

Journals in fields like biology, cell biology, and genetics and heredity, which produce significant quantities of data unsurprisingly often include significantly larger datasets in their SM files, underscoring the critical role of SM in presenting complex datasets. For example, in the like biology category, SM files frequently include extensive gene expression datasets, protein interaction data, and pathway analyses. Similarly, in cell biology, SM is often used to present detailed experimental conditions, imaging data, and quantitative analyses. In contrast, narrative-driven fields, such as public health and nutrition dietetics, tend to have smaller SM data size, using these files primarily for supplemental explanations. This variability underscores the distinct roles that SM files play in different disciplines, emphasizing the importance of tailoring data-sharing strategies to the specific needs of each research field.

### FAIR-SMART enables precise SM data retrieval

The diverse formats of SM files often hinder programmatic searches for articles containing scientific raw data. Instead, users are required to manually navigate each article, review the associated SM files, and extract the desired data – a process that is both time-consuming and labor-intensive. While making SM accessible in a standardized format enhances interoperability, making the information findable is also important for usability and promoting data reuse.

To address this challenge, we use FAIR-SMART to enable efficient search and retrieval of SM data. To identify articles containing relevant SM data in tables, a biomedical text encoder – MedCPT [19] – was used to convert the contents of all tables for each SM file into an embedding vector (768 dimensions). We firstly categorized the SM files into 40 clusters which were defined by ChatGPT via the 10,000 sampled articles based on the cosine similarity from the clusters to each article. Users can simply assign the pre-defined code, listed in S4 Table, to specify the type of resource (e.g., GPED to Gene Expression Data) to search.

To demonstrate the capability of FAIR-SMART in retrieving relevant SM data, we compared it against PubMed, PMC-full text search and the newly released NLM Dataset Catalog (https://www.datasetcatalog.nlm.nih.gov/) using queries spanning 14 most popular SM topics that together cover over 80% of all SM tables. To evaluate its effectiveness in identifying the most relevant SM content, we compared the accuracy (precision) of the top-10 articles retrieved. Our evaluation involved identifying articles containing supplementary resources relevant to the specified types. We manually reviewed the top-10 results and classified the articles as containing either relevant or irrelevant data. The detailed evaluation protocols are attached in the supplementary material.

Some articles returned by PubMed are not part of PMC, which makes bioinformatics workflows more difficult since the data cannot be downloaded programmatically. This issue is further complicated by potential restrictions on data reuse policies. While PMC retrieves full-text articles, its performance is less accurate than PubMed’s. In PubMed results, the query is usually included in the title or abstract, ensuring higher relevance. In contrast, the top results from PMC may only mention the query a few times across different parts of the full text and are not required to include it in the title or abstract. This makes PMC results less relevant compared to PubMed’s top results.

In addition, the NLM Dataset Catalog often redirects users to external databases (e.g., dbGaP [20]), which may require registration or download permissions, creating difficulties for large-scale data mining. PMC and PubMed results may also include articles where relevant data is found in main-text tables rather than supplementary tables. Main-text tables tend to focus on analysis and often provide only summarized information, which may lack the detail needed for comprehensive data reuse.

**Fig 5:**
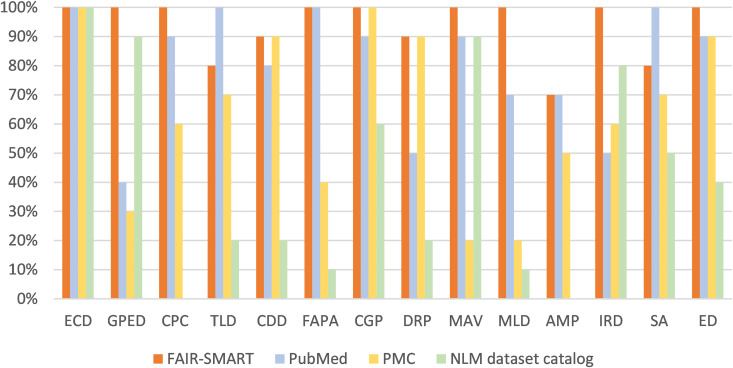
Top-10 precision comparison of FAIR-SMART, PubMed, PMC, and the NLM Dataset Catalog for retrieving supplementary material (SM) data across 14 biomedical topics. The reached articles contain SM data matching the query type. Only precision (i.e., the proportion of relevant articles among the top-10 results) is evaluated; recall or overall coverage is not assessed in this comparison. FAIR-SMART consistently achieves higher precision across all categories, demonstrating its effectiveness in retrieving specific types of SM data. Definitions of the categories: ECD: Experimental Conditions and Designs; GPED: Gene Expression Data; CPC: Cohort and Patient Characteristics; TLD: Experimental Characteristics or Transaction Log Data; CDD: Clinical Diagnostics Data; FAPA: Functional Annotation and Pathway Analysis; CGP: Comparative Genomics and Phylogenetics; DRP: Dose-Response or Pharmacokinetics; MAV: Genomic Mutation and Variant Data; MLD: Metabolomics and Lipidomic Data; AMP: Animal Model Phenotyping; IRD: Immune Response Data; SA: Survival Analysis; ED: Ecotoxicology Data. The numerical data for this Figure can be found in S1 Data.

As shown in Fig 5, FAIR-SMART substantially improves data retrieval, consistently outperforming standard PubMed and PMC relevance and the NLM Dataset Catalog across all queries. For instance, we demonstrated the retrieval of gene expression data. FAIR-SMART enables researchers to efficiently locate and extract SM files containing specific gene expression profiles. FAIR-SMART allows users to automatically identify additional articles with relevant SM, reducing the need for manual inspection and enhancing the coverage in SM data retrieval. To evaluate the coverage of relevant articles, we conducted an additional analysis using three search engines—PubMed, PMC, and FAIR-SMART—across 14 representative SM categories. From the 10 top-retrieved articles by each engine for each category (10 × 3 × 14 = 420 total entries), we manually identified 336 unique articles containing relevant data of the category. Among them, PubMed retrieved 114, PMC retrieved 96, and FAIR-SMART retrieved 129, demonstrating the highest precision and breadth. Notably, only 4 articles overlapped between the PubMed and PMC results, reflecting the divergent indexing and ranking mechanisms of each platform. This diversity in retrieval further highlights the value of FAIR-SMART in offering a complementary and comprehensive search experience. By unifying structured access with semantic relevance, FAIR-SMART not only expands retrieval coverage but also helps accelerate discoveries in fields such as functional genomics, disease mechanism research, and precision medicine.

To further assess the validity of the LLM-generated category groupings used in FAIR-SMART, we analyzed the highest-frequency MeSH terms from SM tables assigned to each of the 14 categories. To improve specificity, we first removed the top 50 general-purpose MeSH terms, identified based on their broad distribution across multiple categories, before identifying category-specific terms. As shown in S5 Table, the remaining top MeSH terms consistently aligned with the semantic focus of their corresponding categories. For instance, “Polymorphism, Single Nucleotide” (MeSH ID: D020641) emerged as a prominent term in the “Mutational Analysis/Variants” category. These results support that the LLM-based grouping strategy effectively captures the underlying topical structure of SM content and enhances the semantic relevance of the retrieved results.

Beyond comparison of the retrieved data quality, it is important to note that the NLM Dataset Catalog primarily aims to provide raw data rather than analyzed data with summaries which provide context. For example, when seeking cohort characteristic information, the data available in PMC main-texts or SM files mainly describe the characteristics of patient groups. In contrast, the data provided by the NLM Dataset Catalog typically consist of raw data for individual patients, lacking summarized cohort-level insights. Furthermore, the NLM Dataset Catalog contains 80,905 datasets (in January 2025), a significantly smaller number compared to the extensive SM data available in PMC Open Access, which includes over 2 million articles with SM files.

In addition to reducing the manual effort required for data extraction, FAIR-SMART ensures the retrieval of highly relevant datasets for diverse biomedical inquiries, enhancing both the efficiency and quality of research workflows.

### Two use cases: Unlocking untapped potential for biomedical discovery

SM data represents a largely untapped resource for expanding biomedical knowledge. However, it is often overlooked due to challenges in findability, accessibility, and interoperability caused by the heterogeneous file formats. These materials provide detailed datasets, experimental protocols, and results that frequently exceed the depth and scope of main text articles. For example, SM datasets include genetic variants [17],pathway analyses [21], and gene sets [22]—critical for understanding complex biological systems.

Genomic variant databases, such as dbSNP [23] and ClinVar [24], rely on manual curation to annotate genomic variants and associated knowledge but are often hindered by challenges posed by heterogeneous file formats. As demonstrated in S2 Table, the number of variants in SM files is approximately 10 times greater than those in full text, highlighting the necessity of collecting annotations from SM files. By standardizing and streamlining the representation of these data, the machine-readable format of FAIR-SMART addresses these challenges, enabling more efficient data integration and curation.

The vast number of gene set analyses generated through omics approaches provide critical insights into biological pathways and networks of cancer and other diseases. However, much of this data remains buried in supplementary materials as figures or tables, making access and reuse challenging and time-consuming. While many databases (e.g., MSigDB [25], NDEx [21]) compile data from existing resources (e.g., KEGG [26], WikiPathways [27], BioCarta [28]), they often overlook raw data in supplementary materials, missing a valuable opportunity to fully harness its potential for advancing biomedical research. By searching for “gene set enrichment analysis (GSEA)” on PubMed and FAIR-SMART within the Gene Expression Data (GPED) data type, we manually reviewed the top 50 articles. We found that 76% of articles retrieved by FAIR-SMART include gene sets, which is significantly higher than the corresponding 26% from PubMed. The detail of the evaluation is provided in S6 Table. This strongly suggests that our resource can substantially improve data collection efficiency and reduce labor-intensive efforts. By leveraging cutting-edge tools which align with FAIR principles, like FAIR-SMART, researchers can unlock the potential of SM data, accelerating advancements in genomics, proteomics, and systems biology.

## Discussion

By integrating SM data into structured text mining workflows via FAIR-SMART, we demonstrated how these resources can be used to systematically address key challenges in biomedical research, particularly in reproducibility and transparency. Our analysis revealed that SM files often contain detailed datasets such as gene expression profiles and experimental conditions, contributing significantly more data than main texts—more than 100 times of the tabular content—underscoring their potential for advanced analyses. Categories like “Gene/Protein Expression Data” and “Experimental Conditions/Designs” dominate SM files, emphasizing their importance for replicating experiments and advancing research.

By standardizing these diverse and data-rich files into BioC-compliant formats, FAIR-SMART enables seamless integration of SM files into automated workflows, as demonstrated by its ability to retrieve relevant datasets with higher precision compared to PubMed’s relevance-based search. This innovation not only improves the accessibility and utility of SM files but also highlights their value as essential resources for disciplines such as genomics, proteomics, and precision medicine. These findings validate the potential impact of FAIR-SMART in addressing the reproducibility crisis and promoting large-scale, data-driven biomedical research.

In contrast, tables included in the main texts serve a different purpose. They are crafted to highlight key findings and present concise summaries of the study’s outcomes, often emphasizing conclusions or essential insights. These tables are designed with the target audience in mind, aiming to communicate the study’s central messages effectively without requiring the audience to navigate through extensive datasets. As such, main text tables strike a balance between informativeness and accessibility, ensuring that the primary takeaways are readily comprehensible to a broader readership.

This difference between the two types of resources (main texts and SM files) highlights their complementary roles. SM files serve as a rich repository of detailed underlying data, catering to those who seek in-depth exploration, validation, or replication of the study’s findings. In contrast, main texts focus on presenting clear, synthesized information to support the narrative of the research, offering conclusions and insights tailored for broader comprehension. Given this dynamic, ensuring that SM data are programmatically accessible becomes a critical task.

One example of this data is high throughput gene expression studies, which produce large, detailed datasets often stored in tabular format. These data-rich tables are integral to SM, as they provide the foundational raw data for analyses like pathway enrichment or differential expression, ensuring reproducibility and transparency in biomedical research.

While FAIR-SMART demonstrates strong utility, we acknowledge the limited validation of some LLM-based components. Our table categories were quality checked via manual review and internal metrics. However, large-scale benchmarking against human annotations and bias assessments remain future work. We plan to develop curated evaluation sets and monitor performance consistency as models and data evolve.

In recent years, some publishers have adopted policies encouraging – or even requiring – data to be deposited in external repositories, where it can be cited independently of the article. While this approach offers benefits for long-term preservation and discovery, meaningful data reuse depends on contextual information, such as detailed descriptions of how the data were collected and processed [29]. Although such information can be uploaded to external repositories, it is typically already present in the publication itself, allowing PMC articles that include supplemental material to preserve this context by taking advantage of the tight coupling between PMC-hosted data and the associated article. Rather than endorsing a specific mode of data sharing, our approach aims to support discoverability and use for the substantial proportion of PMC articles that include supplemental material. By facilitating access to both the data and its surrounding context, our work enables effective text mining and interpretation.

## Conclusions

FAIR-SMART addresses the critical challenges of utilizing SM in biomedical research, transforming them into valuable resources for automated workflows. By standardizing and structuring SM files using the BioC format, FAIR-SMART overcomes the lack of uniformity and structure in diverse file formats, enabling efficient extraction and integration of data. This approach directly tackles the difficulty posed by heterogeneous formats such as PDFs, Excel sheets, and Word documents. To address the challenge of searching SM, FAIR-SMART provides a Web API that supports programmatic access and retrieval of SM data, eliminating the need for manual inspection and significantly reducing the time and effort required to locate relevant datasets. The API also facilitates the discovery of detailed datasets, such as gene expression profiles and pathway analyses, making them accessible for secondary analyses in fields like genomics, proteomics, and drug discovery. Furthermore, by converting SM files into machine-readable formats, FAIR-SMART removes barriers to automation and scalability, enabling the integration of SM data into text mining, NLP, and machine learning workflows. This structured approach allows researchers to handle large datasets from SM across multiple articles, making large-scale analyses and meta-studies feasible.

These advancements demonstrate how FAIR-SMART aligns with the FAIR principles by improving findability, accessibility, interoperability, and reusability of SM data. By providing standardized, structured, and machine-readable formats, this work allows SM datasets to be seamlessly integrated into advanced biomedical research workflows. Despite these advancements, certain limitations remain. Tabular data in PDF files can only be converted into free text, resulting in the loss of table structures. Other resource types, such as audio and images, fall outside the scope of this work, and text within these resources is not processed. Additionally, only SM files in the PMC open-access subset are accessible due to open access policies, which restricts the range of available data. To address these challenges, future efforts will focus on investigating solutions for preserving table structures in PDF files and expanding the capabilities of FAIR-SMART by integrating downstream text-mining tools like PubTator for information retrieval and knowledge discovery on SM data.

Notably, although structural formatting is not preserved for PDF-derived tables, such cases account for only about 2% of all SM tabular data. For use cases that require full formatting, hyperlinks to the original supplementary files are retained and provided via the API, allowing users to directly access the source materials. This ensures that high-fidelity data remains available when structural preservation is critical for downstream applications.

In summary, FAIR-SMART enhances transparency, reproducibility, and scalability in biomedical research by unlocking the vast potential of SM data. This innovative tool not only simplifies the retrieval and analysis of these rich datasets but also paves the way for deeper insights and discoveries in advanced biomedical research.

## Method

### Conversion pipeline for SM data to BioC format

**Fig 6:**
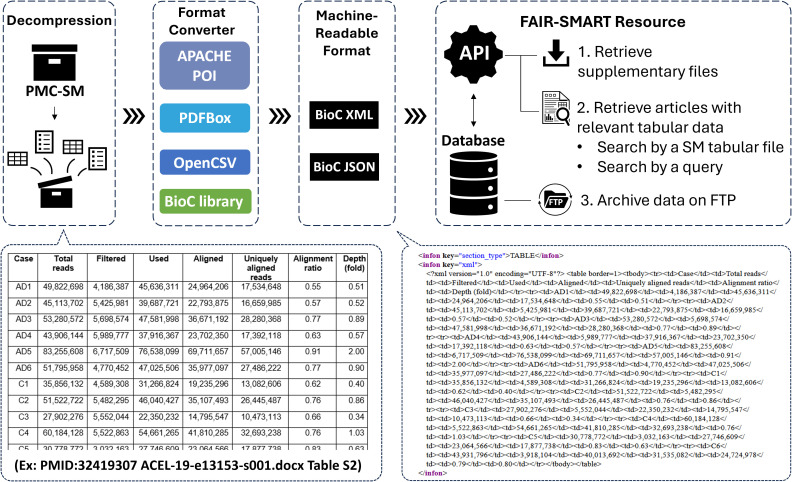
FAIR-SMART Pipeline for unified format conversion of SM files and API usage.

To facilitate the extraction and analysis of SM from PMC Open Access articles, we developed a pipeline for converting textual data from SM files into the BioC format, which is standardized and machine-readable. Fig 6 illustrates the pipeline, which leverages the BioC to organize the rich data found in SM, enabling efficient retrieval and text mining of key biomedical data. The first step in the pipeline involves retrieving the SM files from the PMC Open Access subset. PMC provides articles in structured formats (e.g., XML), but the SM files often use a variety of unstructured formats, including PDFs, spreadsheets, and text files.

To address the heterogeneity of file types, the pipeline utilizes open-source automated text extraction tools. Apache PDFBox (https://pdfbox.apache.org/) is employed for processing PDFs, Apache POI (https://poi.apache.org/) handles Microsoft Office documents (including Word, Excel, and PowerPoint files), and OpenCSV (https://opencsv.sourceforge.net/) is used for comma-separated value (CSV) files. These tools convert diverse file formats into a unified, text-minable output. Additionally, we extracted the contents of compressed archive files and processed the textual files contained within. This approach ensures that key SM files, such as gene expression profiles and clinical trial results, are accurately captured and made accessible for subsequent analysis. The format converter is available at https://doi.org/10.5281/zenodo.17216055.

Following the format conversion step, the SM textual files are transformed into two BioC-compliant formats (i.e., BioC-XML and BioC-JSON) to ensure standardization and compatibility with downstream text-mining and analysis workflows. The original table structures from spreadsheet data are preserved within the XML output, facilitating seamless integration into other text-mining tools or pipelines. However, extracting tabular data from PDF files is extremely challenging because PDFs are designed for visual display rather than structured data storage. Tables in PDFs are often stored as unstructured text and graphics, lacking clear semantic indicators of rows or columns. As a result, in this work, tabular data from PDF files is converted into raw text without preserving the table structure.

As of December 20, 2024, of 5,112,828 SM textual files (including 826,916 textual files within compressed archives), we successfully converted 99.46% (5,085,219 files). The remaining 0.54% of files failed to convert due to various issues, including invalid header signatures, incompatible file versions, and varying degrees of file damage. Additionally, a few hundred converted files could not be imported into the database for API access because their file sizes exceeded 1GB after format conversion (e.g., srep03544-s1.xls in PMC6506566). However, these files are still included in the released archive data available on our FTP site.

To further evaluate the robustness of the converted data, we manually reviewed the textual files of 100 randomly selected articles. Among the 179 SM files (comprising 47 Word files, 36 Excel files, 93 PDF files, 1 text file, and 2 PowerPoint files) associated with these articles, our approach successfully converted all textual content and preserved the table structure (rows and columns) for 65% of tabular data. The remaining 35% of tables lacking structural preservation originated exclusively from PDF files. Many PDFs, especially those without embedded semantic tags, lack clear indicators for defining rows and columns, making it challenging to preserve table structures during conversion. In this work, we treated all PDFs uniformly and converted their tabular data into raw text, which does not retain table formatting.

### The FAIR-SMART API

The goal of FAIR-SMART is to facilitate efficient access to the SM files of PMC Open Access articles, by offering machine-readable formats and features to support biomedical investigations. We make the SM data accessible via a Web API (https://www.ncbi.nlm.nih.gov/research/bionlp/APIs/FAIR-SMART/), which provides two main outputs: (1) the SM file(s) of an article in BioC format, and (2) the articles with relevant SM files given a user query.

The first function allows users to retrieve all standardized SM data associated with a known article. This includes hyperlinks to the original files, the compressed archive, and the textual data in BioC-format. Users can download multiple SM files from multiple articles simultaneously, with a limit of up to 50 PMIDs or PMCIDs per request. Since SM files can be extremely large and difficult to display in a browser, the API deposits files as attachments if their download size exceeds 5 MB.

The second function retrieves a list of articles containing relevant SM files. It supports two input options (shown in S2e and S2f Fig): a contextual query (e.g., ‘ovarian cancer genomic mutation and variant data’) or an SM file with tabular data. To achieve this, we established a systematic pipeline, which illustrates in S2 Fig, to categorize SM tabular files for supporting semantics search.

Initially, the 12,258 SM tables collected in Table 1 were categorized using ChatGPT (S2a Fig), resulting in over 5,000 distinct table types. To balance granularity with practicality, these categories were further grouped, significantly reducing the total number of types. First, we converted all tables to embeddings using the sentence-transformer, MedCPT [19], and applied K-means [30] to group the categories based on their count and the semantic similarity, calculated using the cosine similarity of their embeddings. For reproducibility, we used a local installation of MedCPT-Query-Encoder to generate embeddings, ensuring stable results independent of API changes. To determine the optimal number of clusters for K-means, we employed three measurements: the Elbow Method [31], the Gap Statistic [32], and the Silhouette Score [33]. The Elbow Method was used to analyze the Within-Cluster Sum of Squares (WCSS) and identify the “elbow point” where the decrease in WCSS slows significantly, indicating a balance between cluster compactness and simplicity. The Gap Statistic compared the clustering dispersion with reference distributions, pinpointing the optimal number of clusters where the gap is maximized. Finally, the Silhouette Score evaluated the quality of clustering by measuring how well each point fits within its cluster compared to others, with higher scores indicating better-defined clusters. By integrating the results from these methods (shown in S1 Fig), we identified 40 as the most appropriate number of clusters, as the curves of the three evaluations flatten after this point. This represents a balance between interpretability and maintaining meaningful semantic distinctions among the groups. Recognizing that a small portion (approximately 1%) of the tables could not be categorized to any of the 40 clusters, we adjusted the clustering to 41 clusters, including an “Other” category; however, the “Other” category was excluded from our analysis. We represent each cluster with the most frequent category in the cluster. For example, 34 categories (e.g., Functional Annotation and Pathway Analysis, Metabolic Pathway Analysis/Reactions, and Signal Pathway Enrichment) were grouped together based on semantic similarity. Among these, “Functional Annotation and Pathway Analysis,” is the most frequent and was chosen to represent the cluster. The clusters improve the balance between granularity and practicality, representing the unique characteristics of SM data.

These categorized tables are then embedded using MedCPT, creating embeddings for the 40 types (S2b Fig). Then, we calculated the cosine similarity of each SM table in the PMC open access dataset with the embeddings of 12,258 tables. We then assign each uncategorized table the type of the closest categorized SM table (S2c Fig). To optimize response time, we pre-ranked the relevant data for each SM tabular file and stored the top 1,000 matches in a database (S2d Fig).

The first option (S2e Fig) for retrieving articles with relevant SM files requires a user-provided query. Along with the query, users specify the expected data type (e.g., genomic mutation and variant data), enabling the FAIR-SMART API to narrow the search to articles containing the specified type of SM files. The API filters the retrieved PubMed articles to only those containing SM files of the specified type. Next, each remaining file is converted into an embedding. Articles are then ranked based on the cosine similarity between their embeddings and the query embedding, and the articles with the highest similarity scores are presented to the users. Although the tables usually relate to the query semantically based on the data type, they typically do not contain the exact query tokens. The second option (S2f Fig) allows users to provide an SM tabular file name along with the article identifier (either a PMID or PMCID). The API then returns the 1,000 most relevant SM tabular files.

## Supporting information

S1 Table The portion of the Supplementary material (SM) file types in PMC open access. “#” and “%” of the “SM files” are the numbers and percentages of the SM files in PMC open access. “#” and “%” of the “Articles with SM files” are the numbers and percentages of the PMC articles with the SM files of the type.
(DOCX)

S2 Table Comparison of the Bio-entity Distributions in Main-Texts and SM Files. (DOCX)

S3 Table Distribution of SM data size (in bytes) per article across different journal categories. (DOCX)

S4 Table The 40 clusters of the supplementary material (SM) file types in PMC open access. BioC-SupplMat API requires the code to specify the retrieve data type. The percentage column reflects the proportion of the SM table counts within each type.
(DOCX)

S5 Table The top-14 SM data categories in PMCOA, along with their most frequently occurring representative MeSH terms. Each category groups SM content by thematic focus (e.g., phenotyping, diagnostics, genomics), and representative MeSH terms are in top rank by frequency within that category. The “Example” column provides sample SM entries from PMC articles to illustrate the typical content of each category.
(DOCX)

S6 Table Evaluation of curatable articles containing gene set enrichment analysis (GSEA) data. By searching for “gene set enrichment analysis (GSEA)” on PubMed and FAIR-SMART, we manually reviewed the top 50 articles and categorized them into four types: (1) Y: articles providing gene sets, (2) O: articles providing raw data links (e.g., GEO (Gene Expression Omnibus) ID), (3) N: articles without gene sets, and (4) D: articles related to databases or systems. Only articles categorized as “Y” were considered positive for the evaluation. The search was conducted on January 5, 2025.
(DOCX)

S1 Fig Three measurements, Elbow Method (a), Gap Statistic (b) and Silhouette Score (c), to reach the best number of clusters for the balance between interpretability and maintaining meaningful semantic distinctions among the groups. The numerical data for this Figure can be found in S1 Data.
(TIF)

S2 Fig The API function to retrieve articles with relevant SM data by a user query. (TIF)

S1 Data Raw numbers of the figures. (XLSX)

## Abbreviations

CSV: comma-separated value
GSEA: gene set enrichment analysis
PMC: PubMed Central
SM: supplementary materials
WCSS: within-cluster sum of squares

## References

[1] Roche DG, Berberi I, Dhane F, Lauzon F, Soeharjono S, Dakin R, et al. Slow improvement to the archiving quality of open datasets shared by researchers in ecology and evolution. Proc Biol Sci. 2022;289(1975):20212780. doi: 10.1098/rspb.2021.2780 35582791 PMC9114975

[2] Price A, Schroter S, Clarke M, McAneney H. Role of supplementary material in biomedical journal articles: surveys of authors, reviewers and readers. BMJ Open. 2018;8(9):e021753. doi: 10.1136/bmjopen-2018-021753 30249629 PMC6157527

[3] Munafò MR, Nosek BA, Bishop DVM, Button KS, Chambers CD, du Sert NP, et al. A manifesto for reproducible science. Nat Hum Behav. 2017;1(1):0021. doi: 10.1038/s41562-016-0021 33954258 PMC7610724

[4] Eglen SJ, Mounce R, Gatto L, Currie AM, Nobis Y. Recent developments in scholarly publishing to improve research practices in the life sciences. Emerg Top Life Sci. 2018;2(6):775–8. doi: 10.1042/ETLS20180172 33530668 PMC7289060

[5] Baker M. 1,500 scientists lift the lid on reproducibility. Nature. 2016;533(7604):452–4. doi: 10.1038/533452a 27225100

[6] Greenbaum D, Rozowsky J, Stodden V, Gerstein M. Structuring supplemental materials in support of reproducibility. Genome Biol. 2017;18(1):64. doi: 10.1186/s13059-017-1205-3 28381262 PMC5382465

[7] Comeau DC, Islamaj Doğan R, Ciccarese P, Cohen KB, Krallinger M, Leitner F, et al. BioC: a minimalist approach to interoperability for biomedical text processing. Database (Oxford). 2013;2013:bat064. doi: 10.1093/database/bat064 24048470 PMC3889917

[8] Comeau DC, Wei C-H, Islamaj Doğan R, Lu Z. PMC text mining subset in BioC: about three million full-text articles and growing. Bioinformatics. 2019;35(18):3533–5. doi: 10.1093/bioinformatics/btz070 30715220 PMC6748740

[9] Wilkinson MD, Dumontier M, Aalbersberg IJJ, Appleton G, Axton M, Baak A, et al. The FAIR guiding principles for scientific data management and stewardship. Sci Data. 2016;3:160018. doi: 10.1038/sdata.2016.18 26978244 PMC4792175

[10] Briney KA. Measuring data rot: An analysis of the continued availability of shared data from a single university. PLoS One. 2024;19(6):e0304781. doi: 10.1371/journal.pone.0304781 38838010 PMC11152257

[11] Tarazona-Alvarez B, Zamora-Martinez N, Garcia-Sanz V, Paredes-Gallardo V, Bellot-Arcis C, Lucas-Dominguez R, et al. Open science practices in general and internal medicine journals, an observational study. PLoS One. 2022;17(5):e0268993. doi: 10.1371/journal.pone.0268993 35639752 PMC9154089

[12] Sixto-Costoya A, Lucas-Domínguez R, Aleixandre-Benavent R, Vidal-Infer A. Is sharing datasets the answer to the new challenges of reproductive biology research? Reprod Sci. 2021;28(4):1023–5. doi: 10.1007/s43032-021-00484-8 33594650 PMC7886301

[13] Lucas-Dominguez R, Alonso-Arroyo A, Vidal-Infer A, Aleixandre-Benavent R. The sharing of research data facing the COVID-19 pandemic. Scientometrics. 2021;126(6):4975–90. doi: 10.1007/s11192-021-03971-6 33935332 PMC8072296

[14] Luo L, Wei C-H, Lai P-T, Leaman R, Chen Q, Lu Z. AIONER: all-in-one scheme-based biomedical named entity recognition using deep learning. Bioinformatics. 2023;39(5):btad310. doi: 10.1093/bioinformatics/btad310 37171899 PMC10212279

[15] Wei CH, Allot A, Lai PT, Leaman R, Tian S, Luo L, et al. PubTator 3.0: an AI-powered literature resource for unlocking biomedical knowledge. Nucleic Acids Res. 2024. doi: gkae235 10.1093/nar/gkae235 PMC11223843 38572754

[16] Allot A, Wei C-H, Phan L, Hefferon T, Landrum M, Rehm HL, et al. Tracking genetic variants in the biomedical literature using LitVar 2.0. Nat Genet. 2023;55(6):901–3. doi: 10.1038/s41588-023-01414-x 37268776 PMC11096795

[17] Jimeno Yepes A, Verspoor K. Literature mining of genetic variants for curation: quantifying the importance of supplementary material. Database (Oxford). 2014;2014:bau003. doi: 10.1093/database/bau003 24520105 PMC3920087

[18] Pasche E, Mottaz A, Gobeill J, Michel P-A, Caucheteur D, Naderi N, et al. Assessing the use of supplementary materials to improve genomic variant discovery. Database (Oxford). 2023;2023:baad017. doi: 10.1093/database/baad017 37002680 PMC10066029

[19] Jin Q, Kim W, Chen Q, Comeau DC, Yeganova L, Wilbur WJ, et al. MedCPT: contrastive pre-trained transformers with large-scale PubMed search logs for zero-shot biomedical information retrieval. Bioinformatics. 2023;39(11):btad651. doi: 10.1093/bioinformatics/btad651 37930897 PMC10627406

[20] Tryka KA, Hao L, Sturcke A, Jin Y, Wang ZY, Ziyabari L, et al. NCBI’s database of genotypes and phenotypes: dbGaP. Nucleic Acids Res. 2014;42(D1):D975–9. 10.1093/nar/gkt1211 PMC3965052 24297256

[21] Pillich RT, Chen J, Churas C, Liu S, Ono K, Otasek D, et al. NDEx: accessing network models and streamlining network biology workflows. Curr Protoc. 2021;1(9):e258. doi: 10.1002/cpz1.258 34570431 PMC8544027

[22] Clarke DJB, Marino GB, Deng EZ, Xie Z, Evangelista JE, Ma’ayan A. Rummagene: massive mining of gene sets from supporting materials of biomedical research publications. Commun Biol. 2024;7(1):482. doi: 10.1038/s42003-024-06177-7 38643247 PMC11032387

[23] Phan L, Zhang H, Wang Q, Villamarin R, Hefferon T, Ramanathan A, et al. The evolution of dbSNP: 25 years of impact in genomic research. Nucleic Acids Res. 2025;53(D1):D925–31. doi: 10.1093/nar/gkae977 39530225 PMC11701571

[24] Landrum MJ, Chitipiralla S, Brown GR, Chen C, Gu B, Hart J, et al. ClinVar: improvements to accessing data. Nucleic Acids Res. 2020;48(D1):D835–44. doi: 10.1093/nar/gkz972 31777943 PMC6943040

[25] Castanza AS, Recla JM, Eby D, Thorvaldsdóttir H, Bult CJ, Mesirov JP. Extending support for mouse data in the Molecular Signatures Database (MSigDB). Nat Methods. 2023;20(11):1619–20. doi: 10.1038/s41592-023-02014-7 37704782 PMC11397807

[26] Kanehisa M, Furumichi M, Tanabe M, Sato Y, Morishima K. KEGG: new perspectives on genomes, pathways, diseases and drugs. Nucleic Acids Res. 2017;45(D1):D353–61. doi: 10.1093/nar/gkw1092 27899662 PMC5210567

[27] Agrawal A, Balcı H, Hanspers K, Coort SL, Martens M, Slenter DN, et al. WikiPathways 2024: next generation pathway database. Nucleic Acids Res. 2024;52(D1):D679–89. doi: 10.1093/nar/gkad960 37941138 PMC10767877

[28] Diamant I, Clarke DJB, Evangelista JE, Lingam N, Ma’ayan A. Harmonizome 3.0: integrated knowledge about genes and proteins from diverse multi-omics resources. Nucleic Acids Res. 2025;53(D1):D1016–28. doi: 10.1093/nar/gkae1080 39565209 PMC11701526

[29] Borgman CL. The conundrum of sharing research data. J Am Soc Inf Sci Tec. 2012;63(6):1059–78. doi: 10.1002/asi.22634

[30] MacQueen J. Some methods for classification and analysis of multivariate observations. Proceedings of 5-th Berkeley Symposium on Mathematical Statistics and Probability. University of California Press; 1967.

[31] Thorndike RL. Who belongs in the family? Psychometrika. 1953;18(4):267–76. doi: 10.1007/bf02289263

[32] Tibshirani R, Walther G, Hastie T. Estimating the number of clusters in a data set via the gap statistic. J R Stat Soc Series B: Stat Methodol. 2001;63(2):411–23. doi: 10.1111/1467-9868.00293

[33] Rousseeuw PJ. Silhouettes: a graphical aid to the interpretation and validation of cluster analysis. J Comput Appl Math. 1987;20:53–65. doi: 10.1016/0377-0427(87)90125-7

