# Supplementary material for: FAIR-SMART expands access to supplementary materials for research transparency
Source: PLoS Biol. 2025 Oct 9;23(10):e3003428. doi: 10.1371/journal.pbio.3003428 (PMC12637962; doi:10.1371/journal.pbio.3003428)
Supplement: S1 Table — “#” and “%” of the “SM files” are the numbers and percentages of the SM files in PMC open access. “#” and “%” of the “Articles with SM files” are the numbers and percentages of the PMC articles with the SM files of the type. (DOCX) [file pbio.3003428.s001.docx]

S1 Table. The portion of the Supplementary material (SM) file types in PMC open access. “#” and “%” of the “SM files” are the numbers and percentages of the SM files in PMC open access. “#” and “%” of the “Articles with SM files” are the numbers and percentages of the PMC articles with the SM files of the type.

| File type | suffixes | SM files | | Articles with SM files | |
| --- | --- | --- | --- | --- | --- |
|  |  | # | % | # | % |
| **Files with textual data** |  | 4,285,645 | 73.74% | 2,077,040 | 32.58% |
| PDF | pdf | 1,756,368 | 30.22% | 1,007,829 | 15.81% |
| Word | doc,docx | 1,322,440 | 22.75% | 795,395 | 12.48% |
| Excel | xls, xlsx | 804,851 | 13.85% | 310,893 | 4.88% |
| Text | csv, tsv, txt | 357,530 | 6.15% | 310,508 | 4.87% |
| PowerPoint | ppt, pptx | 44,456 | 0.76% | 23,739 | 0.37% |
| **Files without textual data** |  | 1,173,357 | 20.19% | 503,090 | 7.89% |
| Others | sav, gff and etc | 711,853 | 12.25% | 461,235 | 7.23% |
| Video/Audio/Image | avi, mp3, eps and etc | 461,504 | 7.94% | 135,099 | 2.12% |
| **Compressed files** | zip, gz, tar, rar and etc | 353,155 | 6.08% | 301,042 | 4.72% |
| Total |  | 5,812,157 | 100% | 2,489,681 | 39.05% |
