# Supplementary material for: FAIR-SMART expands access to supplementary materials for research transparency
Source: PLoS Biol. 2025 Oct 9;23(10):e3003428. doi: 10.1371/journal.pbio.3003428 (PMC12637962; doi:10.1371/journal.pbio.3003428)
Supplement: S2 Table — (DOCX) [file pbio.3003428.s002.docx]

S2 Table. Comparison of the Bio-entity Distributions in Main-Texts and SM Files.

| Resource | Number of entities per article | | | | | |
| --- | --- | --- | --- | --- | --- | --- |
|  | Gene | Disease | Chemical | Species | CellLine | Variant |
| Main texts | 55.09 | 96.32 | 86.73 | 50.77 | 4.6 | 2.35 |
| SM files | 217.25 | 89.76 | 95.58 | 56.34 | 8.65 | 21.69 |
