# Supplementary material for: FAIR-SMART expands access to supplementary materials for research transparency
Source: PLoS Biol. 2025 Oct 9;23(10):e3003428. doi: 10.1371/journal.pbio.3003428 (PMC12637962; doi:10.1371/journal.pbio.3003428)
Supplement: S3 Table — (DOCX) [file pbio.3003428.s003.docx]

S3 Table. Distribution of SM data size (in bytes) per article across different journal categories.

| Category | SM data size per article |
| --- | --- |
| Biology | 4,758,519 |
| Cell Biology | 3,820,299 |
| Cell & Tissue Engineering | 3,514,301 |
| Genetics & Heredity | 3,047,011 |
| Chemistry, Organic | 2,820,557 |
| Electrochemistry | 2,701,085 |
| Materials Science, Biomaterials | 2,595,630 |
| Developmental Biology | 2,591,517 |
| Geochemistry & Geophysics | 2,498,752 |
| Chemistry, Inorganic & Nuclear | 2,268,342 |
| Plant Sciences | 2,246,643 |
| Biotechnology & Applied Microbiology | 2,210,519 |
| Chemistry, Physical | 2,135,549 |
| Energy & Fuels | 2,056,898 |
| Neuroimaging | 2,031,001 |
| Biochemical Research Methods | 1,954,254 |
| Chemistry, Applied | 1,763,846 |
| Mineralogy | 1,754,009 |
| Evolutionary Biology | 1,599,775 |
| Mathematical & Computational Biology | 1,505,536 |
| Biochemistry & Molecular Biology | 1,458,037 |
| Chemistry, Medicinal | 1,394,807 |
| Engineering, Biomedical | 1,384,062 |
| Marine & Freshwater Biology | 1,353,404 |
| Oceanography | 1,349,403 |
| Spectroscopy | 1,282,463 |
| Pathology | 1,243,994 |
| Medicine, Research & Experimental | 1,206,515 |
| Nanoscience & Nanotechnology | 1,173,733 |
| Microbiology | 1,111,568 |
| Neurosciences | 1,015,019 |
| Hematology | 985,959 |
| Anatomy & Morphology | 965,807 |
| Virology | 933,300 |
| Parasitology | 907,814 |
| Oncology | 881,976 |
| Fisheries | 845,442 |
| Political Science | 824,435 |
| Immunology | 807,298 |
| Peripheral Vascular Disease | 636,820 |
| Environmental Sciences | 635,978 |
| Integrative & Complementary Medicine | 633,321 |
| Radiology, Nuclear Medicine & Medical Imaging | 629,208 |
| Psychology, Developmental | 616,564 |
| Psychiatry | 609,845 |
| Medicine, General & Internal | 599,552 |
| Tropical Medicine | 585,092 |
| Chemistry, Analytical | 582,048 |
| Medical Informatics | 581,261 |
| Endocrinology & Metabolism | 562,671 |
| Geriatrics & Gerontology | 517,382 |
| Psychology, Experimental | 516,563 |
| Physiology | 484,429 |
| Psychology, Biological | 476,327 |
| Pharmacology & Pharmacy | 465,468 |
| Zoology | 462,708 |
| Cardiac & Cardiovascular Systems | 461,820 |
| Reproductive Biology | 459,143 |
| Respiratory System | 433,028 |
| Gastroenterology & Hepatology | 432,898 |
| Food Science & Technology | 415,265 |
| Hospitality, Leisure, Sport & Tourism | 413,856 |
| Geology | 391,043 |
| Clinical Neurology | 384,510 |
| Psychology, Educational | 381,599 |
| Critical Care Medicine | 380,895 |
| Audiology & Speech-Language Pathology | 377,995 |
| Behavioral Sciences | 368,762 |
| Mycology | 363,985 |
| Rheumatology | 351,355 |
| Allergy | 330,069 |
| Agriculture, Dairy & Animal Science | 315,933 |
| Engineering, Chemical | 313,109 |
| Toxicology | 308,830 |
| Health Care Sciences & Services | 298,017 |
| Ophthalmology | 294,610 |
| Dentistry, Oral Surgery & Medicine | 291,957 |
| Veterinary Sciences | 291,586 |
| Medicine, Legal | 274,120 |
| Obstetrics & Gynecology | 271,095 |
| Transplantation | 251,214 |
| Ornithology | 250,396 |
| Andrology | 248,013 |
| Urology & Nephrology | 240,174 |
| Pediatrics | 233,383 |
| Psychology | 230,889 |
| Orthopedics | 230,867 |
| Infectious Diseases | 230,307 |
| Polymer Science | 221,100 |
| Surgery | 220,648 |
| Public, Environmental & Occupational Health | 214,689 |
| Entomology | 186,958 |
| Nutrition & Dietetics | 181,966 |
| Substance Abuse | 181,449 |
| Health Policy & Services | 174,765 |
| Rehabilitation | 167,387 |
| Anesthesiology | 165,636 |
| Agriculture, Multidisciplinary | 162,114 |
| Otorhinolaryngology | 159,104 |
| Sport Sciences | 143,600 |
| Psychology, Social | 128,564 |
| Psychology, Applied | 122,701 |
| Medical Laboratory Technology | 115,578 |
| Psychology, Clinical | 112,665 |
| Dermatology | 106,686 |
| Women’s Studies | 106,458 |
| Emergency Medicine | 99,094 |
| Primary Health Care | 77,788 |
| Nursing | 73,971 |
| Criminology & Penology | 70,155 |
| Gerontology | 68,655 |
| Sociology | 63,234 |
| Social Work | 62,285 |
| Medical Ethics | 53,155 |
| Education, Special | 48,822 |
