# Supplementary material for: FAIR-SMART expands access to supplementary materials for research transparency
Source: PLoS Biol. 2025 Oct 9;23(10):e3003428. doi: 10.1371/journal.pbio.3003428 (PMC12637962; doi:10.1371/journal.pbio.3003428)
Supplement: S4 Table — BioC-SupplMat API requires the code to specify the retrieve data type. The percentage column reflects the proportion of the SM table counts within each type. (DOCX) [file pbio.3003428.s004.docx]

S4 Table. The forty clusters of the supplementary material (SM) file types in PMC open access. FAIR-SMART API requires the code to specify the retrieve data type. The percentage column reflects the proportion of the SM table counts within each type.

| Category | Code | # Tables | % |
| --- | --- | --- | --- |
| Experimental Conditions and Designs | ECD | 410,979 | 13.41% |
| Gene Expression Data | GPED | 385,835 | 12.59% |
| Cohort and Patient Characteristics | CPC | 306,245 | 9.99% |
| Experimental Characteristics or Transaction Log Data | TLD | 234,471 | 7.65% |
| Clinical Diagnostics Data | CDD | 227,384 | 7.42% |
| Functional Annotation and Pathway Analysis | FAPA | 168,673 | 5.50% |
| Comparative Genomics and Phylogenetics | CGP | 158,934 | 5.19% |
| Dose-Response or Pharmacokinetics | DRP | 132,762 | 4.33% |
| Genomic Mutation and Variant Data | MAV | 114,885 | 3.75% |
| Metabolomics and Lipidomic Data | MLD | 85,313 | 2.78% |
| Animal Model Phenotyping | AMP | 74,742 | 2.44% |
| Immune Response Data | IRD | 71,425 | 2.33% |
| Survival Analysis | SA | 62,034 | 2.02% |
| Ecotoxicology Data | ED | 56,055 | 1.83% |
| Bibliographic Data Compilation | BDC | 50,464 | 1.65% |
| Statistical Analysis and Correlations | SAC | 41,963 | 1.37% |
| Microbiome Composition | MC | 38,149 | 1.24% |
| Qualitative Analysis of Participant Feedback | QAPF | 32,638 | 1.06% |
| Manuscript Structure and Content Recommendations | MSCR | 31,183 | 1.02% |
| Microscopy and Imaging Analysis | MIA | 30,816 | 1.01% |
| Survey Data and Student Perceptions | SDSP | 28,196 | 0.92% |
| Comparative Analysis of Research Output | CARO | 27,485 | 0.90% |
| Chemical Shift Analysis Data | CSAD | 27,216 | 0.89% |
| Species Distribution Data | SDD | 25,486 | 0.83% |
| Patient Experience and Perception Analysis | PEPA | 25,146 | 0.82% |
| Neurophysiological Data Analysis | NDA | 22,817 | 0.74% |
| Geographical Data | GD | 22,240 | 0.73% |
| Functional Mutagenesis Studies | FMS | 21,411 | 0.70% |
| Computational Algorithm Comparison | CAC | 20,470 | 0.67% |
| Computational Biology and Structural Modeling | CBSM | 18,554 | 0.61% |
| Dietary Exposure Analysis | DEA | 17,607 | 0.57% |
| Machine Learning Model Performance Evaluation | MLPE | 17,303 | 0.56% |
| Biomechanical Analysis Data | BAD | 15,370 | 0.50% |
| Bibliographic Data in Plant Biology Research | BDPBR | 15,318 | 0.50% |
| Molecular Dynamics Simulation Data | MDSD | 14,358 | 0.47% |
| Molecular Dynamics Simulation Data | CMPE | 10,531 | 0.34% |
| Material Properties Analysis | MPA | 10,431 | 0.34% |
| Financial and Operational Metrics Analysis | FOMA | 10,331 | 0.34% |
| Surgical Procedure Consensus Data | SPCD | 5,410 | 0.18% |
| Optical Properties of Graphene Oxide Derivatives | OPGOD | 4,909 | 0.16% |
