# Supplementary material for: FAIR-SMART expands access to supplementary materials for research transparency
Source: PLoS Biol. 2025 Oct 9;23(10):e3003428. doi: 10.1371/journal.pbio.3003428 (PMC12637962; doi:10.1371/journal.pbio.3003428)
Supplement: S5 Table — Each category groups SM content by thematic focus (e.g., phenotyping, diagnostics, genomics), and representative MeSH terms are in top rank by frequency within that category. The “Example” column provides sample SM entries from PMC articles to illustrate the typical content of each category. (DOCX) [file pbio.3003428.s005.docx]

S5 Table. The top 14 supplementary material (SM) data categories in PMCOA, along with their most frequently occurring representative MeSH terms. The listed MeSH terms are the highest-ranking by frequency within each category. The “Example” column provides sample SM entries from PMC articles to illustrate the typical content of each category.

| Category | Category Full Name | Representative MeSH Term | Example |
| --- | --- | --- | --- |
| AMP | Animal Model Phenotyping | D004195\|Disease Models, Animal | Supplementary Table i in PMC11370066 |
|  |  | D017753\|Ecosystem |  |
|  |  | D051381\|Rats |  |
| CDD | Clinical Diagnostics Data | D016896\|Treatment Outcome | Table S4 in PMC11142909 |
|  |  | D016032\|Randomized Controlled Trials as Topic |  |
|  |  | D012189\|Retrospective Studies |  |
| CGP | Comparative Genomics/Phylogenetics | D020869\|Gene Expression Profiling | Supplementary Data 1 in PMC8113528 |
|  |  | D019143\|Evolution, Molecular |  |
|  |  | D017422\|Sequence Analysis, DNA |  |
| CPC | Cohort/Patient Characteristics | D012189\|Retrospective Studies | Supplementary data 1.1 in PMC11229496 |
|  |  | D003430\|Cross-Sectional Studies |  |
|  |  | Q000706\|statistics & numerical data |  |
| DRP | Dose-Response or Pharmacokinetics | Q000188\|drug therapy | Supplement Table 1 in PMC3337406 |
|  |  | Q000494\|pharmacology |  |
|  |  | Q000627\|therapeutic use |  |
| ECD | Experimental Conditions/Designs | Q000494\|pharmacology | Supplemental Table S1 in PMC7263196 |
|  |  | Q000187\|drug effects |  |
|  |  | Q000188\|drug therapy |  |
| ED | Ecotoxicology Data | D017753\|Ecosystem | Table S6 in PMC6417698 |
|  |  | D004784\|Environmental Monitoring |  |
|  |  | D003247\|Conservation of Natural Resources |  |
| FAPA | Functional Annotation and Pathway Analysis | D020869\|Gene Expression Profiling | Additional file 12 in PMC8638416 |
|  |  | D059467\|Transcriptome |  |
|  |  | D015398\|Signal Transduction |  |
| GPED | Gene/Protein Expression Data | D020869\|Gene Expression Profiling | Supplementary Data 3 in PMC8791982 |
|  |  | D015972\|Gene Expression Regulation, Neoplastic |  |
|  |  | D005786\|Gene Expression Regulation |  |
| IRD | Immune Response Data | Q000469\|parasitology | Supplementary Table 1 in PMC9792507 |
|  |  | D015658\|HIV Infections |  |
|  |  | D000086402\|SARS-CoV-2 |  |
| MAV | Mutational Analysis/Variants | D020641\|Polymorphism, Single Nucleotide | Supplementary Data 3 in PMC4703835 |
|  |  | D020022\|Genetic Predisposition to Disease |  |
|  |  | D055106\|Genome-Wide Association Study |  |
| MLD | Metabolomics/Lipidomics Data | D055432\|Metabolomics | Supplementary data 1 in PMC6355784 |
|  |  | D055442\|Metabolome |  |
|  |  | D013058\|Mass Spectrometry |  |
| SA | Survival Analysis | D001943\|Breast Neoplasms | S1 Data in PMC7228055 |
|  |  | D011379\|Prognosis |  |
|  |  | Q000401\|Mortality |  |
| TLD | Transaction Log Data | Q000523\|psychology | Table S1 in PMC4973105 |
|  |  | Q000706\|statistics & numerical data |  |
|  |  | D011795\|Surveys and Questionnaires |  |
