# Supplementary material for: FAIR-SMART expands access to supplementary materials for research transparency
Source: PLoS Biol. 2025 Oct 9;23(10):e3003428. doi: 10.1371/journal.pbio.3003428 (PMC12637962; doi:10.1371/journal.pbio.3003428)
Supplement: S6 Table — By searching for “gene set enrichment analysis (GSEA)” on PubMed and FAIR-SMART, we manually reviewed the top 50 articles and categorized them into four types: (1) Y: articles providing gene sets, (2) O: articles providing raw data links (e.g., GEO (Gene Expression Omnibus) ID), (3) N: articles without gene sets, and (4) D: articles related to databases or systems. Only articles categorized as “Y” were considered positive for the evaluation. The search was conducted on January 5, 2025. (DOCX) [file pbio.3003428.s006.docx]

Table S6. Evaluation of curatable articles containing gene set enrichment analysis (GSEA) data. By searching for “gene set enrichment analysis (GSEA)” on PubMed and FAIR-SMART, we manually reviewed the top 50 articles and categorized them into four types: (1) Y: articles providing gene sets, (2) O: articles providing raw data links (e.g., GEO (Gene Expression Omnibus) ID), (3) N: articles without gene sets, and (4) D: articles related to databases or systems. Only articles categorized as “Y” were considered positive for the evaluation. The search was conducted on January 5, 2025.

| Rank # | PMC-OA-BioC | | PubMed | |
| --- | --- | --- | --- | --- |
|  | PMID | Type | PMID | Type |
| 1 | 33719329 | Y | 16199517 | N |
| 2 | 30885118 | D | 30664679 | D |
| 3 | 39003797 | Y | 34174849 | D |
| 4 | 39463421 | Y | 37886446 | D |
| 5 | 19014579 | Y | 35681225 | Y |
| 6 | 38493246 | Y | 36524127 | Y |
| 7 | 38111587 | Y | 36532015 | Y |
| 8 | 39097701 | Y | 37841872 | D |
| 9 | 39314848 | Y | 33719329 | D |
| 10 | 22694750 | Y | 35346191 | O |
| 11 | 39438952 | Y | 37149072 | N |
| 12 | 31517839 | Y | 30885118 | D |
| 13 | 35660007 | D | 36313717 | N |
| 14 | 19725948 | D | 37781041 | N |
| 15 | 28852598 | Y | 37633529 | O |
| 16 | 36159817 | Y | 35669784 | O |
| 17 | 37081490 | Y | 35757739 | N |
| 18 | 33495413 | Y | 35684042 | N |
| 19 | 34789836 | Y | 24962434 | N |
| 20 | 37638029 | Y | 33435872 | D |
| 21 | 37370046 | Y | 36969832 | O |
| 22 | 38928215 | Y | 17644558 | D |
| 23 | 38692177 | Y | 38433839 | Y |
| 24 | 36782176 | O | 18854360 | D |
| 25 | 32117620 | Y | 36830707 | N |
| 26 | 35095893 | Y | 33861629 | D |
| 27 | 28973463 | D | 31638917 | Y |
| 28 | 26481321 | D | 28499413 | D |
| 29 | 29843660 | Y | 17526521 | D |
| 30 | 35095892 | Y | 34429388 | Y |
| 31 | 30018734 | Y | 23632162 | D |
| 32 | 38848448 | Y | 36439178 | N |
| 33 | 33986994 | Y | 39640533 | Y |
| 34 | 37540673 | O | 31307154 | D |
| 35 | 34040895 | O | 32692836 | D |
| 36 | 31229436 | Y | 39084678 | D |
| 37 | 33819193 | Y | 35706447 | D |
| 38 | 31714939 | Y | 38224712 | N |
| 39 | 36518297 | N | 37580667 | O |
| 40 | 35029926 | Y | 27279483 | Y |
| 41 | 38740818 | Y | 38363924 | O |
| 42 | 35535692 | Y | 35873477 | Y |
| 43 | 35059509 | D | 28123101 | O |
| 44 | 36591215 | D | 24884810 | D |
| 45 | 32782440 | Y | 37275904 | D |
| 46 | 26080057 | Y | 35518641 | Y |
| 47 | 33691763 | O | 32316383 | Y |
| 48 | 25880967 | Y | 19645689 | D |
| 49 | 33571282 | Y | 29185027 | Y |
| 50 | 33407928 | Y | 38382096 | O |
| 51 | 31410310 | Y | 17127676 | Y |
