# Supplementary figures and images for: FAIR-SMART expands access to supplementary materials for research transparency
Source: PLoS Biol. 2025 Oct 9;23(10):e3003428. doi: 10.1371/journal.pbio.3003428 (PMC12637962; doi:10.1371/journal.pbio.3003428)

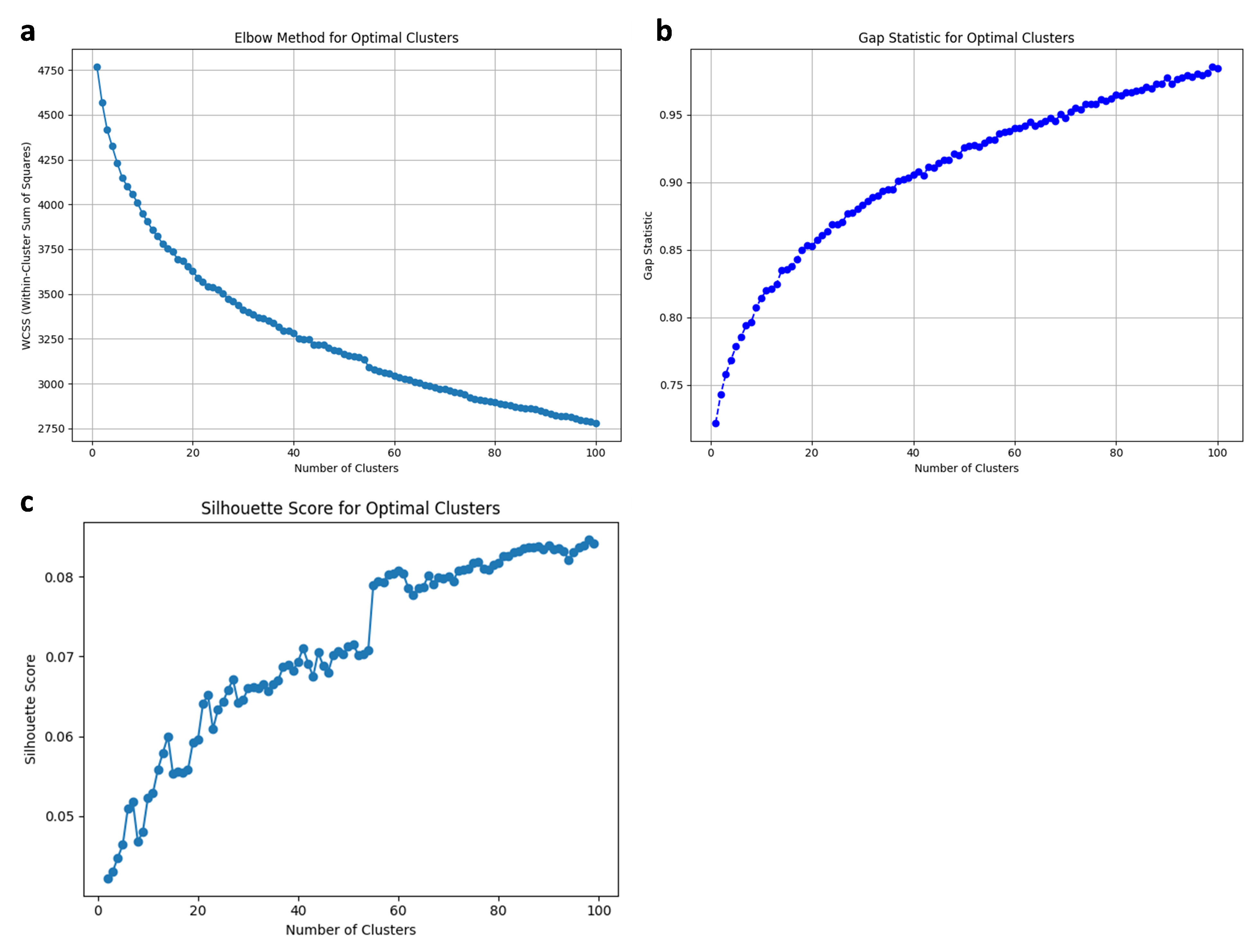

Supplement: S1 Fig — The numerical data for this Figure can be found in S1 Data. (TIF) [file pbio.3003428.s007.tif]

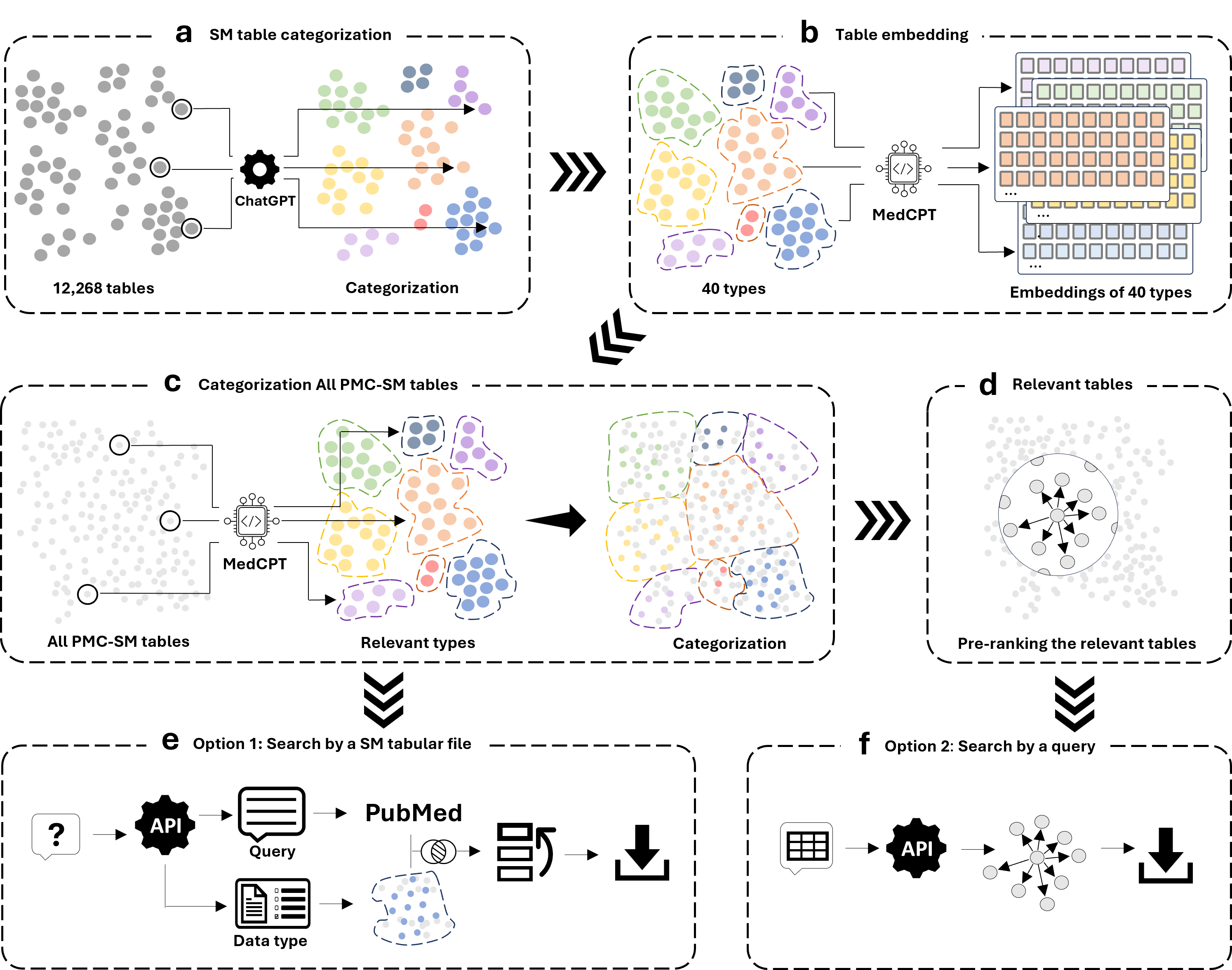

Supplement: S2 Fig — (TIF) [file pbio.3003428.s008.tif]
